# One Click Away: Digital Mentorship in the Modern Era

**DOI:** 10.7759/cureus.1838

**Published:** 2017-11-11

**Authors:** Michael Gottlieb, Abra Fant, Andrew King, Anne Messman, Daniel Robinson, Guy Carmelli, Jonathan Sherbino

**Affiliations:** 1 Department of Emergency Medicine, Rush University Medical Center; 2 Emergency Medicine, Northwestern University Feinberg School of Medicine; 3 Emergency Medicine, The Ohio State University Wexner Medical Center; 4 Emergency Medicine, Wayne State University; 5 Emergency Medicine, The University of Chicago School of Medicine; 6 Emergency Department, SUNY Downstate Medical Center, Brooklyn, NY, USA; 7 Emergency Medicine, McMaster University, Hamilton, ON

**Keywords:** mentorship, digital, professional development

## Abstract

Mentorship is a valuable component of the career development of junior faculty. The digital era has allowed for greater access to mentors spanning geographic barriers and time zones. This article discusses the concept of digital mentorship, as well as strategies and techniques for developing and supporting a digital mentoring relationship in the modern era.

## Introduction and background

The origin of the term ‘mentor’ comes from Homer’s *The Odyssey* [1]. Mentor was the teacher for the son of Odysseus when he went to war. The term mentor was later defined as “a teacher, a role model, an approachable counsellor, a trusted adviser, a challenger, and an encourager” [2]. Mentoring is “a process for the informal transmission of knowledge, social capital, and support perceived by the recipient as relevant to work, career, personal, or professional development” [3]. Since Hippocrates and his followers, mentorship has informed the very origin of medical education via the relationship of master and novice. In modern medicine, mentorship has been shown by multiple studies to have a positive influence, including decreased burnout and depression [4], increased career satisfaction [5], and personal and career development [6,7]. For early career researchers, having a mentor has been shown to improve one’s chances of obtaining research grants and enhancing research performance [5,6,8]. In academia, structured mentoring can be a cost-effective way to improve skills needed for academic success and to promote retention of medical educators in academic centers [9].

Despite the evidence supporting the value of mentorship, some providers have indicated a lack of access to mentors at their institution [6,10]. Faculty development programs have arisen as one pathway to increase the availability of mentorship [11-13]. These programs exist at both the institutional and national level to provide educational resources and mentoring that may be limited in one’s local department. While there has been evidence of benefit with some of these structured programs, they are not universally available at all institutions and may require significant time and monetary commitments [11]. Limitations with schedule availability and office hours can further limit one's access to the mentor.

Additionally, while a mentor may be a valuable resource in one arena (e.g., research), he or she may be a poor mentor in another (e.g., work-life balance). At a minimum, one should target mentorship for each of the following arenas: research skills, promotion and career development, and personal goals [14]. Therefore, one will often need more than one mentor to cover multiple career interests, which can be challenging to obtain at a single institution.

Finally, while local mentors remain important for navigating institution-specific issues and local politics, there may be limited options due to both available expertise and capacity among potential mentors. External mentorship can allow for access to more niche-specific mentors, as well as greater understanding of external options and career pathways to further help career development and satisfaction.

The digital revolution has broken down geographical barriers and facilitated global connections. The current generation of learners seeks help online just as often as they look locally for answers [15]. Both business and academia have identified this trend, leading to the creation of online communities for like-minded learners [16] and web-based platforms to connect geographically-separated mentors and mentees with each other [17-20]. In this paper, we discuss the concept of digital mentorship and suggest strategies and techniques for developing and supporting a digital mentoring relationship in the modern era.

## Review

Strategies for identifying and obtaining a digital mentor

**Figure 1:**
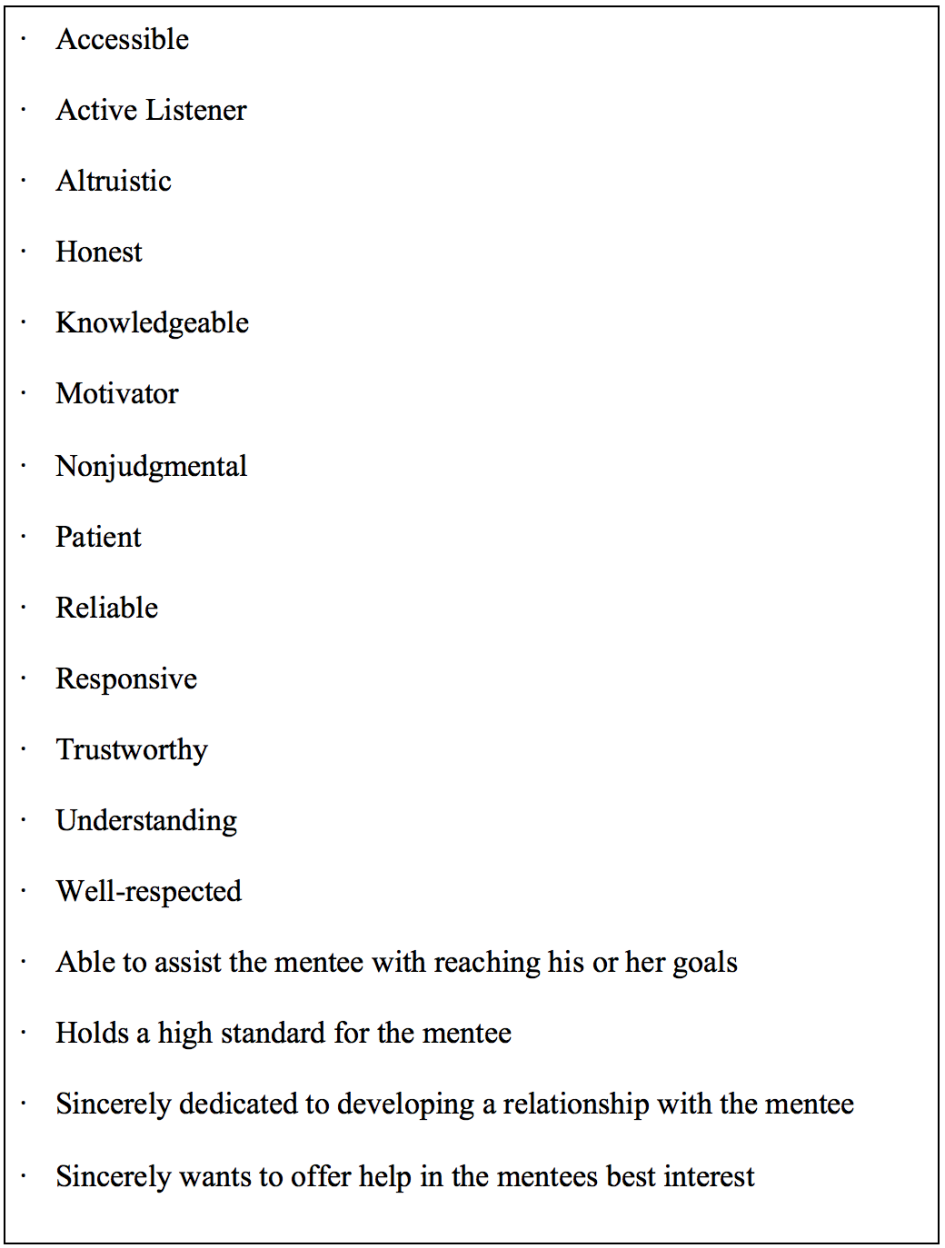
Key Attributes of a Successful Mentor.

Junior faculty who desire a career in medical education must be proactive and persistent in their search for mentorship [21]. With the continued development of technology and virtual communities of practice, mentorship can be identified regardless of geography [22]. Given the benefits of an engaged mentor, junior faculty should set aside time to identify someone who will fill that role successfully (Figure 1). They should select individuals who share similar interests or career paths, or who would serve as a valuable source of advice [22]. Depending upon the junior faculty’s niche, a local mentor may not be the best, or even a feasible option. Having a digital mentor, or an array of mentors from disparate locations around the world, can be a valuable resource [23]. Seeking mentorship must be an active pursuit; however, the mentee may not always know how to begin [24].

A practical way to begin searching for a mentor, either in-person or digitally, is to utilize existing social networks. A junior faculty member could start by speaking with more seasoned faculty within their own department. When attending academic meetings, he or she should approach lecturers that presented material in-line with personal or career interests, and consider emailing potential mentors prior to the meeting [22]. If interested in Free Open Access Medical Education (FOAM) resources such as blogs or podcasts, mentorship may also be found by reaching out to the creators of specific resources. Twitter is another way to identify like-minded individuals and to obtain virtual mentorship. Joining into online discussions is an easy way to increase one’s exposure to potential mentors without ever leaving one’s home or office. The conversations can serve as an opening to continue a conversation online or offline with the potential mentor. Twitter can also be utilized to send a direct message to speak more in-depth with the person, when one does not have the person’s email. Other networking sites (e.g., LinkedIn, Facebook, Doximity, SERMO) allow for users to create online profiles and reach out to potential mentors. As with all online communication, it is important for one to maintain professionalism and remember that anything posted online is available diffusely and indefinitely. This is a unique challenge of the online medium, of which providers should be aware.

The interpersonal aspect of the mentoring relationship is crucial and can represent a problem in institutions that assign mentoring pairs. The mentor and mentee must have compatible personalities and similar professional interests; therefore, a pilot is often necessary to determine if the mentoring relationship should continue [23]. Similarly, when beginning a relationship with a digital mentor, it can be valuable to arrange a face-to-face meeting, either at a national conference or when visiting the mentor’s city to determine whether the mentoring dyad is the correct fit. When this is not possible, a “virtual” face-to-face meeting via the Internet (e.g., Google Hangout, Skype, FaceTime) can allow for an improved connection when compared to email or phone calls by the addition of eye contact and non-verbal communication.

Although existing contacts can make an introduction easier, cold calls can also be an effective means of establishing a mentoring relationship. Creating an electronic elevator pitch is a great exercise for junior faculty to hone their career narrative, and it can serve as a quick introduction to send to potential mentors. An elevator pitch refers to a short, often 30-second-long, description of a person, intended to introduce oneself, provide a brief and compelling background story, and conclude with a directed request (e.g., mentorship, consideration for an employment opportunity, potential research collaboration) [25]. In electronic format, this should be limited to one or two paragraphs of text. Utilizing this exercise for self-reflection may help junior faculty to create more defined goals for a potential mentor-mentee relationship [21]. Often, one mentor may not meet all existing needs of the mentee; therefore, the development of a few pitches to engage mentors in different arenas may be helpful [26].

In fact, in order to address the variety of career development needs of junior faculty members, it may be necessary to have multiple mentors, sometimes referred to as a “personal board of directors” [27]. This can be a tremendous asset because most mentors will not have expertise in all of the areas where educators need advice. Similarly, not all mentors will be available when advice is needed. The development of multiple mentoring relationships provides a breadth of experiences and wisdom to draw upon. Each mentoring relationship is unique, and will provide different conversations, relationships, and learning opportunities [26]. While there are advantages to having multiple mentors, a disadvantage is that each mentor may have differing opinions about the preferred course of action, thus making the mentee’s decisions more difficult [24].

While there is no defined maximum number of mentors, one should focus on identifying a select group of high-quality mentors, rather than aiming for a larger quantity of mentors. As the range of potential mentors expands, it is important to re-evaluate one’s current mentor list and refine the list to those most in-line with the mentee’s personal and professional goals. This can best be performed by evaluating which mentors are most frequently utilized and which advice has been most effective.

Another novel strategy developed from the business world is the concept of the “mastermind group” [28]. This involves developing a group of collaborators who each serve as near-peer mentors to the other group members. The emphasis is on structured meetings with support and targeted feedback. The availability of potential members regardless of geographic boundaries can allow for a much more robust group of mentors and advice.

Techniques for maximizing and maintaining digital mentorship

Digital mentorship, similar to other forms of mentorship, requires work and adaptability [22,24]. The ability to have a long-lasting and fulfilling digital mentoring relationship necessitates that the relationship be nurtured and maintained [26]. Regular contact and following up is key in both digital and in-person mentorship. Many of the challenges, barriers, and ways of improving traditional mentorship experiences (e.g., active engagement, self-awareness, commitment to the relationship) still hold true for digital mentorship [24]. Additionally, while face-to-face meetings may not always be possible, digital mentorship still requires a time commitment from both the mentor and the mentee. Therefore, it is important to establish expectations early on. The mentee must be clear regarding what their career goals are and how the mentor may be of help. When meeting virtually, the mentee must be prepared and on-time [29]. Similar to in-person mentorship, outside distractions (e.g., cell phones, emails) should be minimized during the meeting. Mentorship in the digital age still necessitates that the mentor should help the mentee navigate the professional community (locally and virtually) and point the mentee towards resources if the mentor is unable to help with a certain task [29].

One of the challenges encountered in the digital mentoring relationship is the absence of physical proximity between the mentor and mentee. Similar to in-person mentorship, digital mentees need to be able to have access to their mentors. In local mentoring relationships, both parties reside in the same institution, which can serve as a reminder and natural location for meetings given the proximity to each other. This can be more challenging when the mentor and mentee may be hundreds of miles and several time zones apart. Therefore, it is important for digital mentees and mentors to ensure regular communication, which can be both synchronous (e.g., direct video or voice chat), as well as asynchronous (e.g., email and text messages). Mentees should schedule regular meetings with their mentor and vice versa. Digital meeting options can include video chat via smartphones or computer-based methods (e.g., Skype, Google Hangout), as well as voice-only meetings (e.g., phone calls). National meetings are another excellent option for mentors and mentees to meet face-to-face with some regularity, but should be planned in advance [22]. All virtual or in-person conversations should be followed by written conversation, which can be valuable for outlining the discussion and subsequent plans, as well as providing a reminder of the next steps. When communicating via email or text, it is important for both mentors and mentees to be responsive and timely [30].

Limitations and future directions

Despite the advantages listed above, there are several potential limitations associated with digital mentorship. Because the mentoring relationship can involve distant locations and differing time zones, it may be challenging to maintain the relationship in the same manner as with local mentors who often reside in offices near to one’s own. Additionally, it is important to have local mentors, as well, to help navigate local politics and institution-specific issues. Finally, while digital mentorship can expand one’s potential list of available mentors, it does not substitute for poor mentorship relationships. One should seek out mutually beneficial and invested relationships, which may often involve more than one mentor depending upon the area of interest. Multiple studies have highlighted problematic mentorship relationships, which can include lack of commitment on the part of the mentor, perceived competition, and even conflicts of interest with the potential for abuse of the relationship and stealing of project ideas [31]. These challenges can occur in either local or digital mentorship and one should be cognizant to avoid or end mentoring relationships that demonstrate signs of the above issues. Future studies should assess the satisfaction and effectiveness of digital mentorship. It would also be valuable to develop a national virtual mentor network within the various medical disciplines.

## Conclusions

Mentorship opportunities have evolved significantly with the increasing globalization and access to resources provided by the Internet. This paper provides an overview of digital mentorship, providing specific strategies and techniques for developing and maintaining successful relationships. This paper may help improve virtual mentorship relationships for both learners and faculty.
